# Musical Sophistication and the Effect of Complexity on Auditory Discrimination in Finnish Speakers

**DOI:** 10.3389/fnins.2017.00213

**Published:** 2017-04-13

**Authors:** Caitlin Dawson, Daniel Aalto, Juraj Šimko, Martti Vainio, Mari Tervaniemi

**Affiliations:** ^1^Cognitive Brain Research Unit, Faculty of Medicine, University of HelsinkiHelsinki, Finland; ^2^Phonetics and Speech Synthesis Research Group, University of HelsinkiHelsinki, Finland; ^3^Institute for Reconstructive Sciences in Medicine, Misericordia Community Hospital, University of AlbertaEdmonton, AB, Canada; ^4^Cicero Learning, University of HelsinkiHelsinki, Finland

**Keywords:** quantity language, Finnish, musicality, auditory processing, discrimination, brainstem

## Abstract

Musical experiences and native language are both known to affect auditory processing. The present work aims to disentangle the influences of native language phonology and musicality on behavioral and subcortical sound feature processing in a population of musically diverse Finnish speakers as well as to investigate the specificity of enhancement from musical training. Finnish speakers are highly sensitive to duration cues since in Finnish, vowel and consonant duration determine word meaning. Using a correlational approach with a set of behavioral sound feature discrimination tasks, brainstem recordings, and a musical sophistication questionnaire, we find no evidence for an association between musical sophistication and more precise duration processing in Finnish speakers either in the auditory brainstem response or in behavioral tasks, but they do show an enhanced pitch discrimination compared to Finnish speakers with less musical experience and show greater duration modulation in a complex task. These results are consistent with a ceiling effect set for certain sound features which corresponds to the phonology of the native language, leaving an opportunity for music experience-based enhancement of sound features not explicitly encoded in the language (such as pitch, which is not explicitly encoded in Finnish). Finally, the pattern of duration modulation in more musically sophisticated Finnish speakers suggests integrated feature processing for greater efficiency in a real world musical situation. These results have implications for research into the specificity of plasticity in the auditory system as well as to the effects of interaction of specific language features with musical experiences.

## Introduction

Native language has been shown to influence auditory processing. Mandarin speakers, whose language has lexical tones, show more precise pitch representation in the brainstem and enhanced pitch contour detection in the auditory cortex (Xu et al., 2006; Chandrasekaran et al., 2009; Bidelman et al., 2011). Likewise, Finnish speakers, whose language has a durational (quantity) contrast between long and short in both vowels and consonants, show enhanced duration processing in the form of a smaller just noticeable difference (JND) for duration but not frequency (Tervaniemi et al., 2006), larger mismatch negativity (MMN) amplitude for duration in native speakers of Finnish compared to German speakers or Finnish second-language users (Nenonen et al., 2003; Tervaniemi et al., 2006), and more synchronized brainstem responses when compared to German speakers (Dawson et al., 2016).

Musical training is also known to affect auditory processing, enhancing pitch representation, and temporal precision of subcortical responses, deviant detection, and behavioral frequency and duration discrimination accuracy (Amenedo and Escera, 2000; Kishon-Rabin et al., 2001; Tervaniemi et al., 2005; Rammsayer and Altenmüller, 2006) as well as structural and functional reorganization of cortical areas which is sensitive to different kinds of musical activities (Pantev et al., 1998; Tervaniemi, 2009). These enhancements occur not only for musical stimuli like chords and melodies but also natural and synthetic speech, iterative ripple noise (IRN), and tone bursts (Schön et al., 2004; Wong et al., 2007; Kraus and Chandrasekaran, 2010), and suggest a domain-general enhancement of auditory processing. Supporting this idea, some studies have shown bi-directional influences between music and language (Patel and Iversen, 2007; Bidelman et al., 2011; Kraus and Slater, 2015), suggesting that rather than a one-way effect, there is interaction. These influences have been described as near and far transfer effects which show enhancements for intra-and inter-domain enhancements (e.g., musical training enhancing melodic memory or phonemic skills, respectively) as well as effects specific to musical and linguistic experiences (Moreno and Bidelman, 2014; Strait and Kraus, 2014; Hutka et al., 2015).

It is a well-known psychophysical phenomenon that sound features are not perceived independently; perception is based on systematic interactions of sound features. Sounds that are louder or higher in pitch are generally perceived to be longer than less intense or lower pitched sounds of the same objective duration (Henry, 1948; Pisoni, 1976). These biases are somewhat modulated by native language background, e.g., Finnish speakers are more influenced by pitch when making durational judgments than Mandarin speakers (Aalto et al., 2013; Šimko et al., 2015). While Finnish does not have lexical tone, its quantity contrasts are co-signaled by a downward pitch glide on the longer vowel, suggesting a fundamental link between pitch and duration (Suomi, 2005; Vainio et al., 2010). Marie et al. (2012) found that Finnish nonmusicians had comparable behavioral deviant detection for duration and frequency to French musicians, while the French musicians had larger MMN amplitudes to frequency deviants than either Finnish or French non-musicians. The evidence suggest domain-general processing for sound features in both music and speech. However, it is unclear whether musical training affects these biases within different language populations. Therefore, the current study investigates how these systematic language-modulated biases interact with musicality within a specific quantity language speaking population.

Use of a correlational analysis rather than a cross-sectional comparison of groups was motivated by recent research indicating that the effects of musical *experiences* (not only professional-level training) may occur on a faster and more nuanced scale than can be shown by comparing professional musicians with very nonmusical people. Rapid plastic effects similar to long term training effects have been shown from relatively minimal amounts of training in both music and speech (Menning et al., 2002; Gaab et al., 2006; Song et al., 2008; Tierney et al., 2013). Differences in behavioral and brain responses have been recorded from musicians with different stylistic backgrounds, implying long-term fine-tuning of auditory processing due to music training (Tervaniemi et al., 2012, 2013, 2015; Vuust et al., 2012). There is also some research indicating that musical experiences at different periods of auditory development may affect the auditory system in different ways, and there may even be a critical or at least sensitive period for musical training (Trainor, 2005; Bailey and Penhune, 2012; Skoe and Kraus, 2013), although the extent to which a critical period may impact a spectrum of musicality or musical skills is unclear.

In a larger picture, it useful to include members of the population who do not fall at the extremes of the spectrum since music remains an important social bonding experience and tends to play a role even in the lives of the least musically trained people. Furthermore, individual differences such as genetic predispositions and possible epigenetic factors related to music (Tan et al., 2014; Kanduri et al., 2015; Schellenberg, 2015), motivation toward music, and exposure to different kinds of musical environments may also influence the differences between behavioral and brain responses to musical stimuli and the interactions between musical experience and language experience. This idea is emphasized by using the Goldsmiths Musical Sophistication Index, a measure of musicality that takes into consideration other factors contributing to interactions with music, weighting them along with formal training to gain a more holistic measure of musical sophistication and greater sensitivity to subtle differences in musical abilities (Müllensiefen et al., 2011, 2014).

The current research aims to begin to disentangle the contributions of both native language and musical experience to sound feature processing in brainstem and behavior in a population of Finnish speakers. More specifically, this study intends to investigate possible correlations between behavioral sound feature discrimination abilities, musical sophistication, and auditory brainstem responses to nonspeech sounds in this population. Comparing data from different time scales provides information about the neural organization of these complex effects on sound processing.

For this study, there were several main predictions. First, it was expected that all three simple discrimination tasks would correlate with musical sophistication, i.e., that people with higher musical sophistication scores would have more precise sound feature discrimination for pitch, intensity, and duration individually. Second, it was predicted that the influence of pitch on duration judgments would correlate negatively with musical sophistication, i.e., that people with higher musical sophistication scores would be less influenced by pitch when making duration judgments. This prediction is supported by the evidence that musical experience trains a more precise representation of sound (Wong et al., 2007). Third, it was predicted that higher musical sophistication scores would correlate with a smaller decline in performance in duration discrimination between the simple and complex duration discrimination tasks, showing a greater general ability to ignore distracting sound features like intensity and pitch. This prediction was motivated by previous evidence showing that musicians have an enhanced auditory selective attention and abilities to suppress irrelevant auditory information (Strait and Kraus, 2011; Kaganovich et al., 2013). Fourth, it was predicted that higher musical sophistication scores would correlate positively with peak amplitude in the auditory brainstem response (ABR), representing a more precise onset response due to duration training from music. Additionally, a negative correlation between ABR peak amplitude and simple discrimination task scores would indicate enhancement in both behavioral and brain levels of precision in sound feature discrimination due to musical experience, i.e., with increase in peak amplitude, simple discrimination task scores should be more precise. Finally, it was predicted that the onset peak delay of the brainstem response would be affected by stimulus intensity (strong vs. weak), and musical experience, as evidenced by the literature (Neely et al., 1988; Musacchia et al., 2007).

## Materials and methods

### Participants

Forty four participants took part in the experiment. Four participants were excluded from all analysis at the beginning due to mild to moderate hearing loss discovered during audiometry screening, leaving 40 participants (31 females, mean age 24.7 years) for analyses. All participants took part in both sections of the experiment; however, 12 participants were excluded from EEG analysis during preprocessing due to retaining less than 50% usable data (see Analysis for exclusion criteria) and two were excluded from behavioral processing due to technical errors in the behavioral data collection. EEG data from 28 participants were analyzed, all right-handed native Finnish speakers (22 females, mean age 24.6 years). Behavioral data from 38 participants were analyzed (29 females, mean age 24.7 years).

Participants were recruited by student email lists within the University of Helsinki, from local Facebook groups for students, and word of mouth. They self-reported using only Finnish language in the first 15 years of everyday life and were screened for normal hearing (thresholds ≤ 25 dB SPL) using an Oscilla USB-350SP audiometer circum-aural headset with automatic pure tone test. The experiment was conducted according to the ethical guidelines of the Declaration of Helsinki and the study protocol was approved by the Committee for Ethical Review in the Humanities and Social and Behavioral Sciences at the University of Helsinki. Participants gave written informed consent before the experiment and were compensated for their time.

### Stimuli

**Figure 1:**
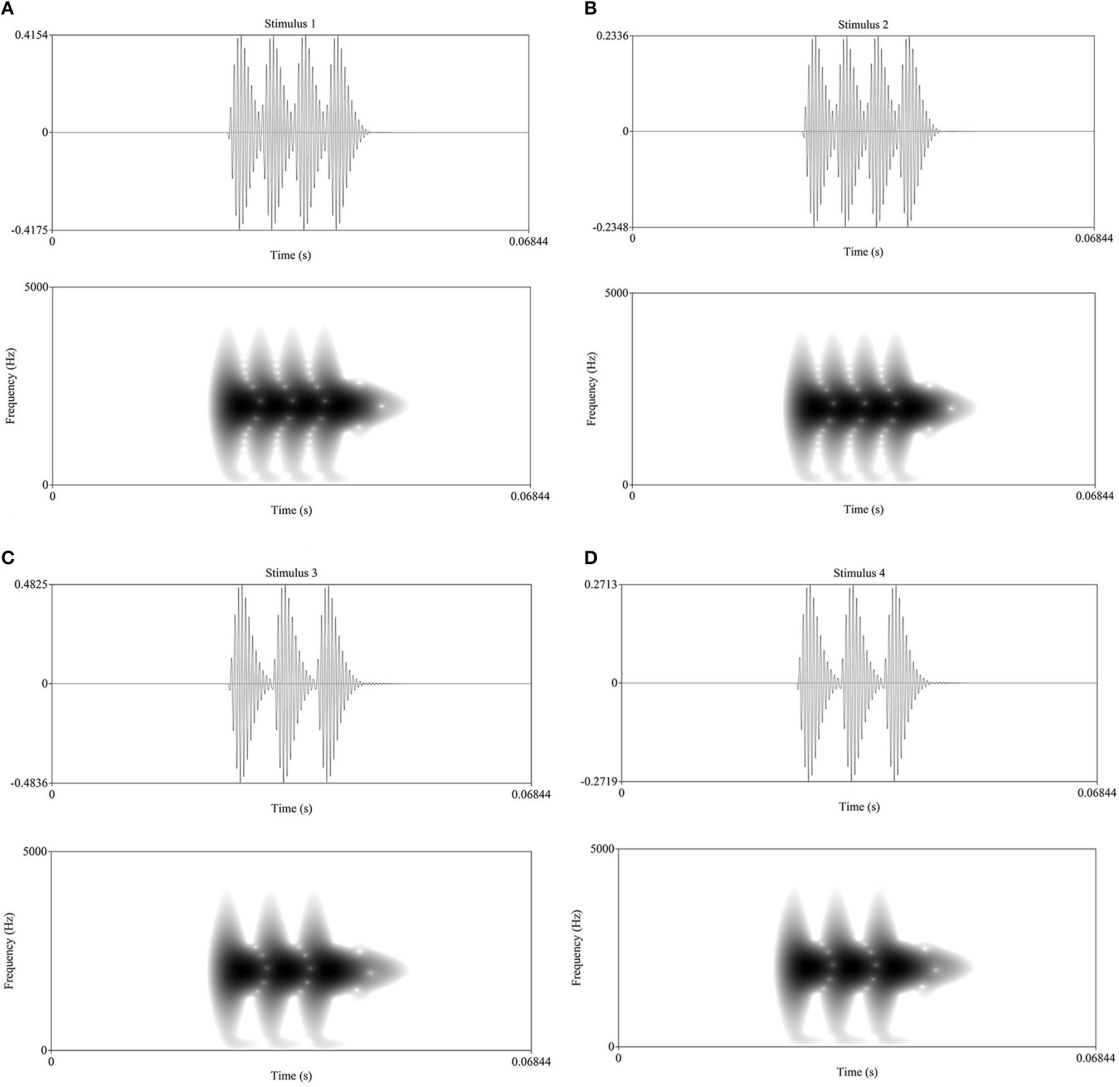
**(A–D)** Waveforms and spectra of the stimuli (positive polarity). “High” stimuli **(A,B)** are 216 Hz; “low” stimuli **(C,D)** are 162 Hz. “Strong” stimuli **(A,C)** are 65 dB (SPL); “weak” stimuli **(B,D)** are 60 dB (SPL).

In the ABR section, there were four narrowband gamma-filtered stimuli which represented intensity-frequency pairings: high-strong, high-weak, low-strong, low-weak. The high frequency stimuli were 162 Hz and low were 216 Hz. The “weak” intensity stimuli were at 60 dB (SPL) and “strong” intensity were 65 dB (SPL) (Figure 1). These stimuli were synthesized in order to maintain strict control over their properties, intended to stimulate a narrow population of neurons while remaining in the frequency range of human speech. A sawtooth wave of each pitch was narrow band filtered using a fourth order polynomial gammatone filter with center frequency 3141.56 Hz; then, average intensities were normalized and the weak stimuli were scaled 5 dB weaker. Each stimulus is about 25 ms in length with a 25 ms silent buffer before and after the sound for an interstimulus interval (ISI) of about 50 ms. It should be noted that these lengths are not actually absolute since the duration of the stimuli depend somewhat on the periodicity of the frequencies, i.e., they are not arbitrary. The peak detection algorithm used in analysis works around this issue by searching for peaks within a defined time window. The pitch sensation created by these short stimuli should reflect the timing properties of the system since individual ABRs are averaged; more synchronized timing would show as higher peaks. Short stimuli were preferred in order to have sufficiently many repetitions while still including multiple stimuli, as consistent differences between the stimuli serve to validate the method. These stimuli are part of a large project investigating sound feature processing in different languages (see also Šimko et al., 2015).

The behavioral stimuli are synthesized in the same way but are longer in order to allow perceptual judgments. The behavioral tasks are created within custom Matlab functions to be within the range of human speech syllables in intensity, frequency, and duration. These three features are either held constant or varied adaptively or randomly, depending on the task.

### Procedure

#### Brainstem recordings

All participants completed the brainstem portion first in order to reduce state differences from boredom, fatigue, or sleeping (Skoe and Kraus, 2010). The entire session (including setup, EEG, behavioral tests, breaks) took approximately 3 h per participant. The ABR portion of the experiment was conducted using a Biosemi 2 Active system, AD rate 16384, with a vertical montage of 10 scalp electrodes placed along the midline channel locations (FPz, AFz, Fz, FCz, Cz, CPz, Pz, POz, Oz, Iz), 2 electrooculography (EOG) electrodes placed at the temples to record saccadic eye movements, and 2 EOG electrodes placed above and below the left eye to record blinking. Two mastoid electrodes were used as references. Participants listened to stimuli at standard 65 dB SPL presented binaurally using shielded circumaural Sennheiser HD 250 linear II headphones in a soundproof, electrically shielded cabin while attending to a silent self-chosen film with Finnish subtitles for four blocks totaling 56 min of EEG recording.

#### Behavioral tasks

In the behavioral section, there were four two-alternative forced-choice tasks. Participants heard two sounds played over circumaural headphones from a laptop calibrated to 65 dB SPL and were asked to choose which sound was louder (Intensity Test), higher (Pitch Test), or longer (Duration Test). Each of the single-feature tests' sounds were varied only in the test dimension and kept constant in the other dimensions. The fourth (multi-feature) test was a Duration Modulation task in which the sounds were varied in all three dimensions simultaneously while the task again asked which sound was longer. The single-feature tests were custom adaptive one-up three-down tests designed to converge on an accuracy level of 75% from Aalto et al. (2013) modified from Kaernbach (1991), and had a maximum of 51 reversals, while the duration modulation task included 300 randomly selected sound pairs. Thresholds were calculated using logistic regression since stimuli in the multi-feature task are randomized and it would not have been possible to use a different thresholding approach, e.g., last 20 reversals, etc. Participants were given as much time as they needed, but each of the single-feature tasks lasted about 10 min, while the complex task typically lasted 20 min.

Musical sophistication scores were gathered according to the self-report musicality questionnaire from the Goldsmiths Musical Sophistication Index (Gold-MSI) (Müllensiefen et al., 2014). Analyses used the generalized score which is further detailed in the Analysis section.

### Analysis

For measures of musicality, the current study uses the self-report questionnaire from the Goldsmiths Musical Sophistication Index (Gold-MSI) (Müllensiefen et al., 2011), which in its full form includes a battery of listening tests including melodic memory, beat perception, and sound similarity; the self-report questionnaire alone has been validated using objective listening tests and is an effective measure of musical ability (Müllensiefen et al., 2014). The self-report inventory scores participants along five factors of musical engagement: active engagement, perceptual abilities, musical training, singing abilities, and emotional engagement. The inventory weights each of these factors together to form a generalized musical sophistication score. The main usefulness of the Gold-MSI is that it controls for individual differences in quality of musical experience, such as the intensity and type of training or genre preferences, and takes into consideration non-training related factors like emotional and social engagement with music, ability to hear and follow pitches and beats (which may or may not be related to professional musical training), motivation toward music, recreational use of music, and whether or not participants enjoy music. Therefore, it is a useful tool for quantifying musical sophistication from both formal training and informal musical experience.

For the behavioral analysis, estimates from a logistic regression model were fitted to the binary response data to calculate the Weber fractions representing each participant's discrimination ability for each sound dimension, with the equation *ln(3)/k* where *k* is the GLM estimate. For the duration modulation test, generalized Weber fractions use the same calculation and represent the extent to which duration is judged longer, given an increase in the specific sound feature (duration, pitch, loudness). Additional ratios were calculated: the intensity ratio, which is the (absolute value of the) ratio of generalized Weber fractions for the intensity dimension over the duration dimension and represents the extent to which participants were influenced by variation in intensity when making the duration judgment (a larger ratio corresponds to more influence), the pitch ratio, which is the same calculation as the intensity ratio but for pitch influence, and duration ratio, which is the ratio of Weber fractions of duration discrimination from the simple task to the complex task, representing the difference in performance between the simple and complex tasks (a smaller ratio corresponds to decrement in performance from simple to complex task). It is expected that all participants decrease in performance due to the added distraction of variation in several sound dimensions at once, which involves more cognitive resources to ignore in order to make the duration judgment. A small number of these ratios were negative, indicating that those participants showed the opposite of the expected discrimination effect direction, e.g., they perceived louder stimuli as *shorter* rather than *longer*, as expected. This likely reflects natural variation in the population or a misunderstanding on the part of the participant. Because of this, the absolute values were used in analysis since the current questions involve the *extent*, and not the *direction*, of duration modulation.

For the ABR analysis, data was preprocessed with band-pass filters at 80 and 4, 000 Hz and an artifact rejection threshold of 30 μV. The participants with less than 50% epochs left after applying this threshold were discarded from analysis. Waveforms were averaged for each stimulus per participant from a random subset of 6,000 epochs (out of a possible total 12,000 with 3,000 sweeps per stimulus with alternating polarities). The subsetting was done in order to resolve the confound of epoch number affecting peak amplitude, since averages over more epochs have a higher signal-to-noise ratio and there is a high variability in data quality between ABR participants. Peak amplitudes and latencies were extracted from this subset with a custom Matlab thresholding algorithm designed to detect peaks within a designated time window as a percentage of total peak size, a conservative measure to take noise into consideration. Peak amplitudes and latencies were then used in correlations with the music scores and behavioral difference limens.

Ten main correlational comparisons were performed: seven for behavioral data and three for brainstem data, Bonferroni corrected for multiple comparisons. In the behavioral data only, correlations were tested between music scores and simple duration discrimination, complex duration discrimination, simple pitch discrimination, pitch ratio, duration ratio, intensity ratio, and simple intensity discrimination. Since the data distributions are non-normal and there is no evidence that musical sophistication would be linearly related to sound feature discrimination, all correlations use Spearman *rho*. Using the brainstem responses, correlations were tested between music score and onset peak amplitude and latency, and between simple duration discrimination and peak amplitude.

Additional linear mixed effects models were fitted to check differences in peak latency and amplitude between stimuli and correlations were tested between musicality score and peak latency.

## Results

### Musicality

Gold-MSI generalized scores for the present study closely matched the distribution of those in the published norms (Müllensiefen et al., 2011) and followed a normal distribution curve with mean score 75.86, maximum 120, and minimum 27. These statistics did not differ between the participants used for ABR data processing and those used for behavioral data processing.

### Behavioral results

**Figure 2:**
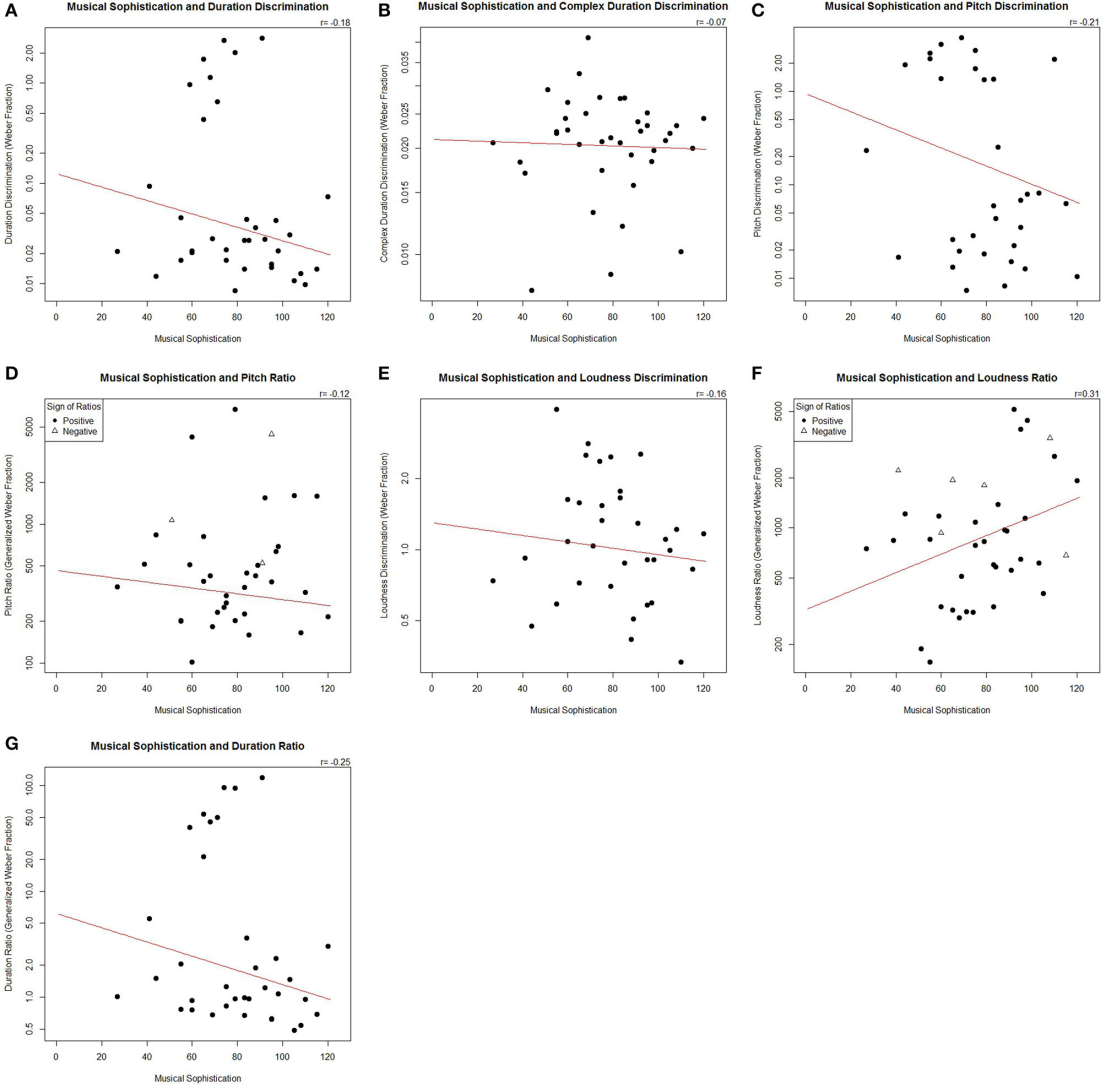
**(A–G)** Scatterplots showing each comparison with behavioral data. Filled circles represent data points from positive values; empty triangles represent originally negative generalized Weber fractions in the complex task.

Seven correlational analyses were performed on the behavioral data (Table 1, Figure 2). Negative Weber fractions and generalized Weber fraction values were transformed to absolute values, which did not noticeably impact the results compared to non-transformed fractions. There were significant correlations between musical sophistication score and simple pitch discrimination, (*S* = 1,640,700, *r*_s_ = −0.21, *p* = 0.017) and between music score and duration ratio (*S* = 1,741,400, *r*_s_ = −0.25, *p* = 0.0024). There was also a correlation between music score and loudness ratio (*S* = 1,343,400, *r*_s_ = 0.31, *p* = 1.25 × 10^−5^). Four correlations with music score were not significant: simple duration discrimination, pitch ratio, simple loudness discrimination, and duration discrimination in the complex task.

**Table 1:** **Correlations performed on behavioral data (Spearman's ***rho***)**.

**Behavioral comparisons**	***r_s_***	***S***	***p***
music × simple duration	−0.18	1,646,900	0.067
music × complex duration	−0.07	2,086,200	*p* > 1
music × duration ratio	−0.25	1,741,400	0.0024^*^
music × simple pitch	−0.21	1,640,700	0.017^*^
music × pitch ratio	0.12	1,724,900	0.58
music × simple loudness	−0.16	1,768,000	0.13
music × loudness ratio	0.31	1,343,400	1.25 × 10^−5^^*^

* *Indicates significant p-values*.

*Absolute values are used for negative fractions*.

More musically oriented people showed enhanced simple pitch discrimination, decreased duration discrimination accuracy between the simple and complex tasks, and a greater influence of loudness on duration judgments in the complex task. However, more musically sophisticated participants did *not* show strong enhancement of simple discrimination for duration or loudness, and musical sophistication did not show a patterned relationship to the influence of pitch on complex duration judgments. Rather, musical sophistication is associated with enhanced simple pitch discrimination alone and a greater decrement in performance in complex duration judgments.

### Brainstem results

There were three comparisons involving the brainstem data: onset peak amplitude with musical sophistication score, onset peak latency with musical sophistication score, and onset peak amplitude with simple duration discrimination. None of these comparisons were significant (*p* > 0.1), and the lack of strong correlation found here between musical sophistication and onset peak latency may suggest that musicality does not confer a substantial onset detection advantage for Finnish speakers.

**Figure 3:**
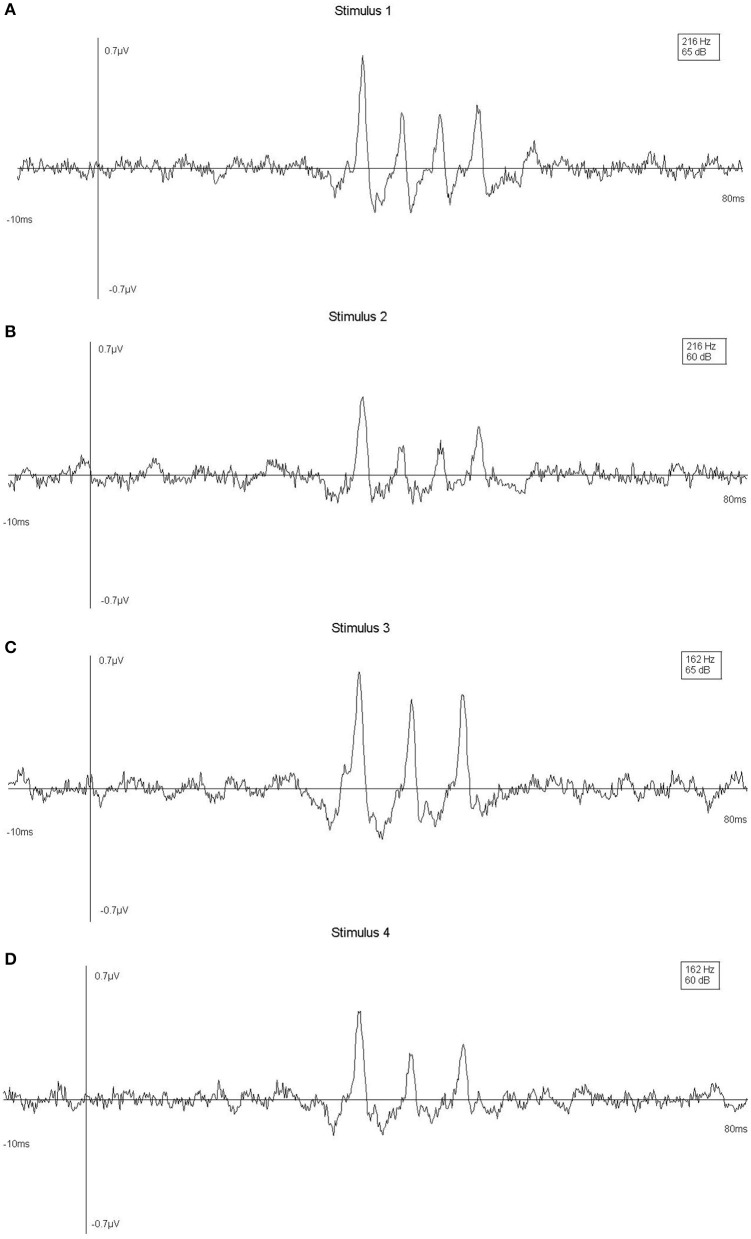
**(A–D)** Averages over trials at A6 (Cpz) channel for one typical participant, by stimulus, showing stimulus effects.

In order to validate the brainstem data, linear mixed effects models were fitted for peak amplitude and latency with stimulus as fixed effect and participant as random effect. Both models showed significant effects of stimulus, in predicted directions. The stronger intensity stimuli showed higher amplitudes and shorter latencies compared to the weaker stimuli (Figure 3), in corroboration with the literature (Eggermont and Don, 1980; Elberling et al., 2010).

For brainstem measurements, the physiological difference in head circumference is a confounding variable often conflated with gender (Mitchell et al., 1989; Aoyagi et al., 1990). A measure of individual hearing threshold was taken as an average between the right and left ear at 4, 000 Hz as recorded by an audiometer. In a linear mixed effects model with music score, hearing threshold, and head circumference as fixed effects and subject as random effects, none of the fixed effects were significant on the peak latency or amplitude of the brainstem response. Therefore, failure to show a relationship here between music score and the brainstem response is not due to the potentially confounding variables of slight differences in hearing threshold nor head size.

## Discussion

In this contribution, we aimed to find out whether associations between musical sophistication and subcortical auditory processing can be observed and, further, whether these processes reflect the known phonological properties of the linguistic background of the participants. Musical sophistication was measured with the General Sophistication score of the self-report inventory, which is part of the Goldsmiths Musical Sophistication Index. The validity of the Gold-MSI has been extensively tested. The self-report inventory scores are correlated with the results of the objective listening tests (which was not used) and shows good internal validity and test-retest reliability (Müllensiefen et al., 2014). Because it includes measures of musical experience which are not necessarily represented by formal training (casual practice and music listening, etc.), as well as taking formal training into the weighting of the factors, it is well suited to both musicians and non-musicians as well as people who may not fall into these categories which are defined by the extent of formal training.

In this population of Finnish speakers, higher musical sophistication scores were correlated with more precise simple pitch discrimination, but on the contrary, pitch influence in the complex duration task was not related to musical sophistication. Musical sophistication scores were also not correlated with performance on the complex duration task alone; however, they were correlated with the ratio of performance on the simple to complex duration discrimination task, i.e., musical sophistication is associated with an intensified decrement in performance with the addition of irrelevant features (less accurate discrimination in the complex task). Musical sophistication was also correlated with the loudness ratio, which means that more musically experienced people were more influenced by intensity when making duration judgments in the complex task. However, the simple loudness judgments were not related to musicality.

It is interesting that there was no correlation of pitch ratio to musicality score. This might imply that there is no pattern for perceiving higher pitch as longer or shorter, based on musicality, that is, more musical Finnish speakers are not more or less likely to judge high pitches as longer (or shorter) than less musical Finnish speakers. The prediction was that more musical sophistication would be related to greater ability to ignore irrelevant features in discrimination tasks. However, it has been shown that the bias toward pitch influence on duration judgment that is present in the general population is relatively strong in Finnish speakers (Šimko et al., 2015), e.g., they are more affected by pitch when making duration judgments than Mandarin speakers. It is possible that musical sophistication changes this bias in a way that would not show an effect with simple correlations in a single-language study, or alternatively, that this bias is not modifiable by musicality but depends more on the language and individual variability.

Although these results may seem counterintuitive, there may be an explanation that relates to expertise and efficiency in the auditory system. It was surprising to find that musicality was not correlated with an overall enhancement of sound features in all three simple tasks. While it is possible that the sample size did not allow detection of a small effect over statistical noise, a likely explanation for the results is that a ceiling effect has been reached. Since adult native Finnish speakers with no language difficulties have reached functional expertise in their language, their auditory pathways are already attuned to processing the subtle duration contrasts in Finnish language. It is possible that this enhancement has reached the zenith of physiological processing power and musical abilities have no space to enhance it further. Therefore, while more musical Finnish speakers show no strong behavioral or electrophysiological enhancement for *duration* processing, music was associated with enhanced *pitch* processing. Since Finnish does not have lexical tones, the intense pitch training from musical experiences was able to confer a specific advantage.

Language-specific adaptation is not necessarily an unusual phenomenon; when comparing successful Cantonese word learning (a language with lexical tones), Thai speakers did not have an advantage over English speakers even though they had experience with a tone language, and Thai musicians did not have a significant advantage over Thai nonmusicians (Cooper and Wang, 2012). English speaking nonmusicians had the best opportunity for enhancement given their non-ceiling tone discrimination abilities and their musically attuned auditory pathways, and they outperformed the other three groups. Additionally, it seems that the Thai speakers were at a slight disadvantage given that their experience with their own native tone language interfered with learning a new one. Their tone-confusion patterns showed that they were more attentive to pitch contour than the English speakers and therefore tended to confuse the gliding tones. The ceiling effect combined with an interference effect from the first language and feature discrimination enhancement from music created a pattern where the effect of musical experience could confer the most benefit to English-speaking musicians. This fits into a new theory of metaplastic enhancement from music; that is, the idea that musical experience, while enhancing specifically trained feature processing, also confers the advantage of training the brain to learn. It has been suggested that this effect could interact with other environmental and innate factors and underlie many of the discrepancies between studies showing different effects of musical training (Merrett et al., 2013).

Similarly, it is possible that in the current experiment, while musical experience enhances the processing of sound features, it also allows for attentional capture when those features are used as distractors which take up cognitive resources to process. Musical experience promotes efficient cortical integration of features in a complex sound environment (Tervaniemi and Brattico, 2004). In the present study, the more musical participants' enhanced ability to analyze pitch may interfere with the accuracy of their duration judgments and corroborates the idea of a combination ceiling-interference effect on a lower, perceptual level. Integration would enhance the accuracy of processing incoming musical stimuli in a complex real-world situation, especially in the case of active performers who are required not only to process sound features efficiently, but also to respond to them appropriately. This practical phenomenon might also explain the correlation of loudness ratio to musical score since in the Western classical style, duration and intensity are tightly linked, especially in expressive performance (Sloboda, 1983). However, this more efficient, integrated top-down processing stream may slow and interrupt the quick perceptual separation of individual features.

Assuming that top-down interference would account for the observed results, we would expect an opposing pattern of enhancement in the single-feature task and influence in the complex task, i.e., enhanced single-feature discrimination is linked to enhanced integration but degraded single-feature discrimination within a complex task. In the complex task, pitch influence on duration judgments showed no interaction with music scores. More musical participants had enhancement in pitch but not loudness discrimination from the single-feature task, so those features which were discriminated more accurately alone (pitch) were not in themselves distracting in the complex task. Loudness, on the other hand, did not show an increase in accuracy with more musical training, and therefore *did* have a direct influence on complex duration processing. Overall, the more musically sophisticated Finnish speakers experienced an increase in interference of the other sound features which prevented them from having a music-based advantage in judging duration in the complex task.

Finally, there is likely a simple reason that there were no correlations involving the brainstem data. The main hypothesis for the brainstem data was motivated by recent research showing a group difference between Finnish speakers and German speakers in amplitude of the brainstem response, indicating an enhanced temporal synchrony (Dawson et al., 2016). In the previous work, simple duration discrimination data was unavailable, and comparisons were made between language groups, so it was not possible to test individual duration discrimination thresholds. The current experiment mainly asks whether the brainstem-level temporal enhancement for duration processing that was observed in Finnish speakers is associated with or further enhanced by the extent of musical sophistication within the language group, as well as the impact of simultaneous processing of different sound features on duration discrimination. Surprisingly, from the behavioral data, it is evidently *not* the case that more musical Finnish speakers experience greater temporal enhancement, and therefore it would not be expected to find a correlation in the brainstem data either. These findings are interesting when considered together since they suggest that enhancements from native language and musicality are not clearly additive.

An additional methodological challenge was a large amount of individual variation in the data. For the single-feature adaptive tasks, there were 51 reversals (from correct to incorrect, and vice versa) allowed, ensuring that the psychometric curves could be well-fitted. The presence of some negative values in the generalized Weber fractions shows that the typical direction of the modulation biases (people tend to judge higher pitches as longer) does not always hold true. A likely explanation for this is an overcompensation in the strategies used by participants (Šimko et al., 2015).

The primary focus of the experiments reported here was duration processing. Because of the observed pitch enhancement, it is reasonable to then ask whether the brainstem data likewise shows an enhanced frequency following response. However, since the main *a priori* hypothesis was related to duration and synchronous processing, the design focused on clear onset responses. The stimuli used here created a pitch sensation with a few cycles but were too short for sustained frequency following response analysis. Therefore, that question was not possible to test here and it remains a compelling point for future research.

The next question will be to ask if this hierarchy of language experience over musical experience applies to other types of languages, namely, tone languages. If the pattern holds, it would be expected that tone language speakers are saturated for pitch, i.e., they would not show an enhancement in simple pitch discrimination associated with musical sophistication, but they would show a duration enhancement associated with musical sophistication. Similarly, native speakers of English, which has neither quantity nor tone, should show enhancement in both dimensions with an increase in musical sophistication. This phenomenon may be related to the many previous findings that show cortical and subcortical enhancement in English speaking musicians.

These findings may be useful for applications for children with language impairments. While current research shows that musical activities can improve children's listening and language skills, it is not yet known whether musical activities targeted toward specific language features may be even more helpful, for example in benefiting phonological awareness and training auditory features to boost processing of specific lexical contrasts (Moreno and Bidelman, 2014). This current research then may contribute toward explaining why musical activities have been shown to help children with language-specific deficits. In the future, these therapeutic activities can be better structured for different types of languages.

## Conclusions

In a population of adult Finnish speakers, musical experience including both formal training and informal engagement with music is correlated with enhanced behavioral pitch discrimination, but not duration discrimination. Greater musical sophistication is also correlated with a more intense decrement in behavioral duration discrimination performance on a complex task including distracting sound features, compared to single-feature tasks. These results can be explained by a ceiling effect set by language, in which the primary lexical contrast encoded in the language (duration for Finnish speakers) is already enhanced, and the feature not encoded in the language (pitch) is able to be enhanced proportionally to musical sophistication. Musical training has been tacitly assumed to enhance sound processing overall, i.e., enhancing processing of different sound features similarly and acting similarly on people from different language groups. However, the current results emphasize that even casual training in specific sound environments can shape the function of the auditory pathway. Changes in this pathway can promote integration and boost efficiency, but at a cost: enhanced processing still consumes cognitive resources and may even interfere with new information. Importantly, musical experience does not necessarily enhance processing of all sound features equally. Instead, this may suggest a hierarchy of plasticity, where language, which is acquired first and is more crucial in terms of social behavioral goals, is primary. Musical experiences are also able to fine-tune the auditory system, importantly, in ways that interact with the native language phonology.

## Author contributions

CD and DA designed the experiment. The behavioral tests were developed by DA, JŠ, and MV. CD collected and analyzed data and wrote the paper. DA, JŠ, MV, and MT edited the paper.

## Funding

The research leading to these results has received funding from the Auditory Cognitive Neuroscience Erasmus Mundus Student Exchange Network, European Community's Seventh Framework Programme (FP7/2007–2013) under grant agreement no 287678 (Simple4All), and a Finnish Academy postdoctoral funding for the third author.

### Conflict of interest statement

The authors declare that the research was conducted in the absence of any commercial or financial relationships that could be construed as a potential conflict of interest.
